# The *Yersinia pestis* Effector YopM Inhibits Pyrin Inflammasome Activation

**DOI:** 10.1371/journal.ppat.1006035

**Published:** 2016-12-02

**Authors:** Dmitry Ratner, M. Pontus A. Orning, Megan K. Proulx, Donghai Wang, Mikhail A. Gavrilin, Mark D. Wewers, Emad S. Alnemri, Peter F. Johnson, Bettina Lee, Joan Mecsas, Nobuhiko Kayagaki, Jon D. Goguen, Egil Lien

**Affiliations:** 1 UMass Medical School, Program in Innate Immunity, Division of Infectious Diseases and Immunology, Department of Medicine, Worcester, Massachusetts, United States of America; 2 Centre of Molecular Inflammation Research, Department of Cancer Research and Molecular Medicine, Norwegian University of Science and Technology, Trondheim, Norway; 3 UMass Medical School, Department of Microbiology and Physiological Systems, Worcester, Massachusetts, United States of America; 4 Department of Medicine, School of Medicine, Duke University, Durham, North Carolina, United States of America; 5 Davis Heart and Lung Research Institute, Division of Pulmonary, Allergy, Critical Care and Sleep Medicine, Department of Internal Medicine, Wexner Medical Center, The Ohio State University, Columbus, Ohio, United States of America; 6 Department of Biochemistry and Molecular Biology, Kimmel Cancer Center, Thomas Jefferson University, Philadelphia, Pennsylvania, United States of America; 7 Mouse Cancer Genetics Program, Center for Cancer Research, National Cancer Institute, Frederick, Maryland, United States of America; 8 Department of Physiological Chemistry, Genentech, Inc., South San Francisco, California, United States of America; 9 Department of Molecular Biology and Microbiology, Tufts University School of Medicine, Boston, Massachusetts, United States of America; University of Pennsylvania, UNITED STATES

## Abstract

Type III secretion systems (T3SS) are central virulence factors for many pathogenic Gram-negative bacteria, and secreted T3SS effectors can block key aspects of host cell signaling. To counter this, innate immune responses can also sense some T3SS components to initiate anti-bacterial mechanisms. The *Yersinia pestis* T3SS is particularly effective and sophisticated in manipulating the production of pro-inflammatory cytokines IL-1β and IL-18, which are typically processed into their mature forms by active caspase-1 following inflammasome formation. Some effectors, like *Y*. *pestis* YopM, may block inflammasome activation. Here we show that YopM prevents *Y*. *pestis* induced activation of the Pyrin inflammasome induced by the RhoA-inhibiting effector YopE, which is a GTPase activating protein. YopM blocks YopE-induced Pyrin-mediated caspase-1 dependent IL-1β/IL-18 production and cell death. We also detected YopM in a complex with Pyrin and kinases RSK1 and PKN1, putative negative regulators of Pyrin. In contrast to wild-type mice, Pyrin deficient mice were also highly susceptible to an attenuated *Y*. *pestis* strain lacking YopM, emphasizing the importance of inhibition of Pyrin *in vivo*. A complex interplay between the *Y*. *pestis* T3SS and IL-1β/IL-18 production is evident, involving at least four inflammasome pathways. The secreted effector YopJ triggers caspase-8- dependent IL-1β activation, even when YopM is present. Additionally, the presence of the T3SS needle/translocon activates NLRP3 and NLRC4-dependent IL-1β generation, which is blocked by YopK, but not by YopM. Taken together, the data suggest YopM specificity for obstructing the Pyrin pathway, as the effector does not appear to block *Y*. *pestis*-induced NLRP3, NLRC4 or caspase-8 dependent caspase-1 processing. Thus, we identify *Y*. *pestis* YopM as a microbial inhibitor of the Pyrin inflammasome. The fact that so many of the *Y*. *pestis* T3SS components are participating in regulation of IL-1β/IL-18 release suggests that these effects are essential for maximal control of innate immunity during plague.

## Author Summary

Many pathogenic Gram-negative bacteria express type III secretion systems (T3SS) that translocate bacterial proteins into host cells with the potential of altering normal cell processes. *Yersinia pestis*, the causative agent of plague, harbors a T3SS which is particularly effective in suppressing innate immunity and release of pro-inflammatory cytokines IL-1β and IL-18, potent triggers of anti-bacterial responses. These cytokines are produced via processing by active caspase-1 in inflammasome complexes. Pyrin is an inflammasome component that recognizes alterations in certain host cell signals. Here we show that the T3SS effector protein YopM inhibits effector YopE-mediated Pyrin-induced caspase-1 activation, IL-1β, IL-18 and cell death triggered by *Y*. *pestis*. We also found that blocking the Pyrin pathway is important for disease development in a mouse model of bubonic plague. Thus, YopM is a microbial molecule blocking Pyrin inflammasomes.

## Introduction

Type III secretion systems (T3SS) are essential virulence factors of many pathogenic Gram-negative bacteria. These systems include a needle-like structure, translocon proteins that form a pore with which the needle can dock in the membrane of host target cells, and a set of secreted effector proteins delivered to the target cell cytoplasm through the docked needle. The effector proteins exert control over key cellular processes that contribute to antibacterial defenses or pathogenesis, including immune signaling, phagocytosis, and induction of cell death. In response, the innate immune system has evolved the ability to recognize a number of T3SS components and initiate protective inflammatory responses when they are detected. In some T3SS-dependent pathogens that cause severe disease—like *Y*. *pestis*, the causative agent of plague—the balance between these opposing activities strongly favors the bacteria.

As we and others have shown, a key strategy of *Y*. *pestis* is preventing production of active IL-1β and IL-18 through an apparent combination of activities [1,2,3,4,5,6]. Maturation of these major pro-inflammatory cytokines is primarily dependent on processing by the protease caspase-1. In turn, activation of pro-caspase-1 depends on assembly of multiprotein intracellular complexes known as inflammasomes, triggered by recognition of the bacterial products or activities via NLR proteins or other alternative pathways. Although the fully intact T3SS of *Y*. *pestis* with its seven secreted Yersinia outer protein (Yop) effectors (YopM, E, K, J, T, H and YpkA) blocks caspase-1 activity effectively, some components of this system are themselves inflammasome activators if the system is incomplete [2,3,7,8], able to trigger anti-bacterial effects [2,5,9]. Thus, to be effective in regulating inflammation, the T3SS must suppress the effects of the same pro-inflammatory signaling systems that it activates. We believe that this small effector toolkit, heavily dedicated towards immune evasion and conferring high virulence [10], makes *Yersinia* an excellent model for characterizing T3SS functions as well as host immune pathways.

In the absence of all seven secreted effector proteins, *Y*. *pestis* producing the T3SS needle and pore-forming translocon pore proteins (YopB, D) activates the NLRP3/ NLRC4 inflammasome pathways effectively, possibly by hypertranslocation of T3SS pore and rod components [3,11]. This activation is blocked by addition of the effector YopK, which can regulate influx of Yops [3,11]. The effector YopJ triggers a non-canonical RIP1-caspase-8-caspase-1 inflammasome pathway [7,12], and also can inhibit NF-κB, MAP2K and MAP3K, reducing synthesis of pro-IL-1β/IL-18. The activation of caspase-8 by YopJ also triggers apoptosis. Loss of YopJ in combination with loss of a second effector, YopM, results in high levels of active caspase-1 and IL-1β/IL-18, comparable to that seen with a strain lacking all seven effectors [1]. YopM was originally proposed to be a caspase-1 inhibitor [4], although an alternative model for YopM inhibition of caspase-1, involving other proteins, has recently been proposed [13]. The precise action of YopM on caspase-1 activation is thus unclear.

Here we report that *Y*. *pestis* YopM is unable to inhibit T3SS-triggered caspase-1 activation mediated by NLRP3, NLRC4, or caspase-8. Instead, this effector inhibits another signal occurring through a Pyrin-dependent pathway. Pyrin (also called MEFV, TRIM20 or marenostrin) is the founding member of the pyrin domain family of proteins. A number of mutations in human Pyrin have been reported and associated with the most common human autoinflammatory disease, Familial Mediterranean Fever (FMF), where the pathology is believed to be initiated by hyperactivation of Pyrin-Asc-caspase-1 inflammasomes [14,15,16]. Bacteria can also activate Pyrin inflammasomes. It was recently proposed that covalent modifications of RhoA GTPase by bacterial toxins and type 6 secretion systems (T6SS), resulting in RhoA inhibition, triggering activation of Pyrin-mediated production of mature IL-1β/IL-18 [17]. YopM is the first specific microbial inhibitor of this incompletely understood pathway to be reported. We also present evidence that the *Y*. *pestis* effector YopE, a Rho inhibitor and GTPase activating protein (GAP), triggers Pyrin inflammasomes. We suggest that inhibition of this pathway by YopM is a central feature of inflammasome suppression observed during *Y*. *pestis* infection.

## Results

### YopM inhibits the Pyrin inflammasome, which is activated by YopE

**Fig 1:**
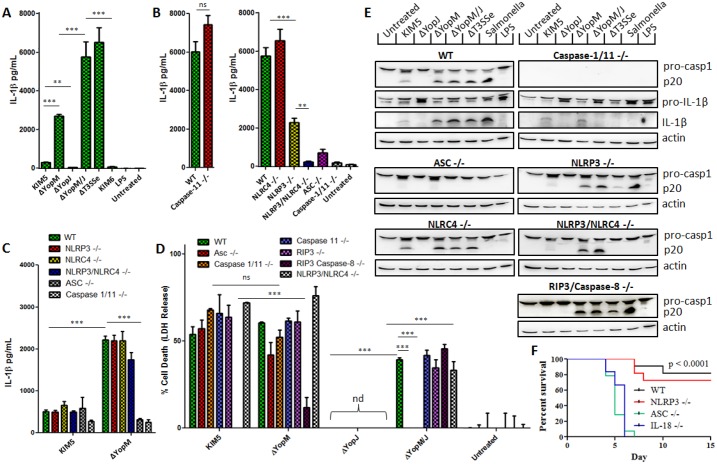
The *Y*. *pestis* effector YopM suppresses a different IL-1β-producing pathway than the one triggered by the needle/translocon through NLRP3 and NLRC4. IL-1β in supernatants from A) WT LPS-primed BMDMs infected with *Y*. *pestis* Yop mutant strains, B) BMDMs of indicated genotypes infected with ΔT3SSe, or C) LPS-primed BMDMs infected with KIM5 and ΔYopM were measured by ELISA 6 hrs p.i. (MOI 10). D) Cell death was assayed by LDH release in LPS-primed BMDMs infected with indicated strains at 6 hrs p.i. (MOI 10). Figures are representative of three or more experiments. E) Total protein from LPS-primed BMDMs infected with indicated strains (combined cell lysate and supernatant) was separated by SDS-PAGE and analyzed by Western Blot for IL-1β and caspase-1. F) Mice of indicated genotypes were injected s.c. with 160 CFU of KIM1001ΔM/J and monitored for survival past 21 days. P value for survival comparisons reflect differences between WT (n = 11) or NLRP3 KO (n = 11) and IL-18 (n = 6), Asc KO (n = 14). Shown is mean plus s.d. for triplicate wells. ND: not detected. A-E are representative of three experiments or more, F representative of two experiments performed. * p<0.05, **p<0.01, ***p<0.001.

Many of the Yersinia T3SS effectors have inhibitory effects on immune functions. *Y*. *pestis* YopM is considered a suppressor of innate immunity, although mechanisms by which it acts are not clear. Both we and others have shown that YopM is an inhibitor of caspase-1 activation [1,4,6,13]. A YopM deletion in *Y*. *pestis* KIM5, normally expressing a fully functional T3SS encoded on the pCD1 plasmid, triggers increased levels of active caspase-1 and IL-1β in mouse primary bone-marrow derived macrophages (BMDM), indicating that YopM suppresses a bacteria-triggered inflammasome pathway, and an additional deletion of YopJ further increases IL-1β release (Fig 1A, [1]). The KIM6 strain, which does not harbor pCD1 and thus lacks the entire T3SS, triggered minimal IL-1β release. In contrast, we observed a strong IL-1β signal in response to the ΔT3SSe strain, lacking secreted effectors but expressing the basic T3SS machinery such as rod/needle/translocon components (Fig 1A). Both NLRP3 and NLRC4 partially contributed to sensing the presence of T3SS needle/translocon (Fig 1B), in addition to caspase-1 and the adaptor Asc. To identify the pathway inhibited by *Y*. *pestis* YopM, we tested whether BMDMs lacking specific inflammasome components would fail to increase IL-1β production when YopM is absent (Fig 1C). Although YopM inhibits a pathway dependent on Asc and caspase-1 (Fig 1C, [1]), we observed no decrease of IL-1β in cells lacking NLRP3, NLRC4, or caspase-11 compared to wild-type cells after infection with *Y*. *pestis* lacking YopM. We also tested cells lacking NLRP12, RIP3, or caspase-8 (S1 Fig), and found none of these proteins to be required for the IL-1β producing pathway which YopM suppresses. Many factors influence bacterial triggering of inflammasomes via T3SS, the stimulation conditions utilized in this project differ from an earlier publication [2] and may not robustly favor activation of the NLRP12 pathway. At present we do not have evidence for how the NLRP12 pathway is triggered and how it interacts with the T3SS-mediated pathways discussed in this manuscript. The same pattern was observed for cell death (Fig 1D); YopM inhibits caspase-1 dependent cell death (pyroptosis) [1], but this cell death still occurs in the absence of the inflammasome components tested above (Fig 1C).

Furthermore, we performed experiments directly comparing effects on caspase-1 cleavage (Fig 1E). YopJ suppresses pro-IL-1β and pro-caspase-1 production [1,6], and triggers a relatively small amount of IL-1β processing in a caspase-8-dependent manner [7]. We noted that caspase-1 may not be absolutely required for caspase-8 dependent IL-1β processing in response to parental *Y*. *pestis* KIM5 (Fig 1E), arguing that caspase-8 can act independently to cleave IL-1β following YopJ action. Caspase-1 activation by *Y*. *pestis* expressing the needle/translocon, but lacking all secreted effectors (ΔT3SSe) is fully dependent on NLRP3/NLRC4, while YopM inhibits caspase-1 processing and IL-1β release independently of NLRP3, NLRC4, NLRP12, RIP3, or caspase-8 (Fig 1E, S1 Fig). It is however a noteworthy point that in NLRP3/NLRC4 deficient cells, *Y*. *pestis* ΔYopM/J triggers a substantial amount of caspase-1 and IL-1β processing while ΔT3SSe (which additionally lacks the other 5 translocated Yops) does not. Taken together, these results indicate that YopM inhibits an Asc-dependent inflammasome triggered by another Yop effector. We have reported that deletion of both YopM and YopJ in a fully virulent *Y*. *pestis* KIM1001 strain implicates increased IL-1β and IL-18 *in vivo*, and leads to significant attenuation following subcutaneous (s.c.) infection mimicking bubonic plague [1]. Here we report that this attenuation is dependent upon Asc but not NLRP3 (Fig 1F), consistent with our *in vitro* data.

**Fig 2:**
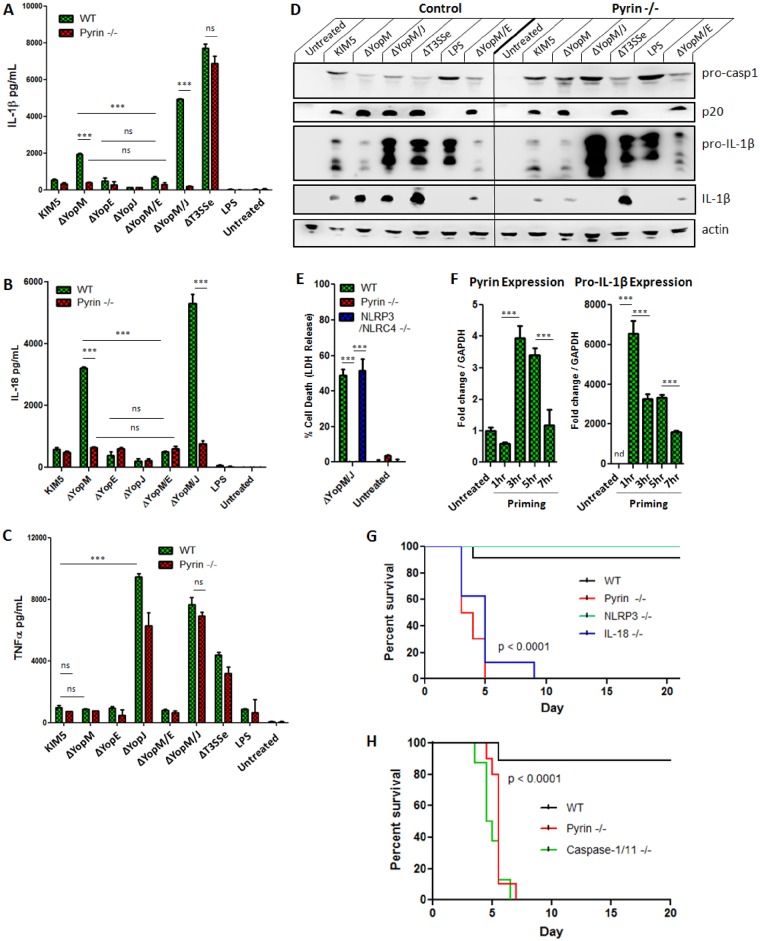
LPS-primed BMDMs were infected with indicated *Y*. *pestis* strains for 6 hours; A) IL-1β, B) IL-18, and C) TNFα were measured in supernatants by ELISA 6 hrs p.i. (MOI 10); D) Total protein from LPS-primed BMDMs infected with indicated strains (combined cell lysate and supernatant) was separated by SDS-PAGE and analyzed by Western Blot for IL-1β and caspase-1. E) Cell death was assayed by LDH release in LPS-primed BMDMs infected with indicated strains at 6 hrs p.i. (MOI 10). F) Expression of Pyrin and Pro-IL-1β mRNA was measured by RT-PCR at 1, 3, 5, or 7 hours after addition of 100ng/mL LPS to WT BMDMs. G) WT C57Bl/6 (n = 12), Pyrin KO (n = 10), NLRP3 KO (n = 7) or IL-18 KO (n = 8) or H) WT (n = 9), Pyrin KO (n = 10) or caspase-1/11 KO (n = 8) mice were infected s.c. with *Y*. *pestis* KIM1001 ΔYopM/J (150 CFU) and monitored for survival up to 21 days. G, H): P value reflects comparison of WT vs Pyrin KO, NLRP3 vs Pyrin KO, WT vs IL-18 KO or WT vs caspase-1/11 KO. Figures are representative of three or more experiments, G, F are representative of two experiments. Shown is mean plus s.d. for triplicate wells. * p<0.05, **p<0.01, ***p<0.001.

We previously suggested YopE as a potential activator of the YopM-inhibited pathway, as removing YopE from the KIM5 ΔYopM strain abolished all the IL-1β blocked by YopM [1]. One key feature of YopE is that this effector inhibits Rho family GTPases via its inherent GAP activity [18,19]. It should be noted that some macrophage anti-bacterial responses induced by YopE have been suggested to be dependent upon its GAP mimetic ability [20]. One remaining candidate for a participant in an Asc-dependent YopM-inhibited inflammasome is Pyrin [21], which has been linked to anti-bacterial innate immunity following RhoA GTPase covalent modification and inhibition [17]. When we tested wild-type BMDMs and BMDMs lacking Pyrin, we found that the IL-1β and IL-18 induction inhibited by YopM is fully dependent on Pyrin, and appears to be triggered by YopE (Fig 2A and 2B). This is comparable to the pathway driving IL-1β induced by *Clostridium difficile* toxin TcdB (S2 Fig). By contrast, TNFα secretion was not appreciably impacted (Fig 2C). Caspase-1 activation (Fig 2D) and pyroptosis (Fig 2E) associated with ΔYopM, in particular when the caspase-8 activating effector YopJ was additionally deleted, were strongly reduced in the absence of Pyrin. Thus, we propose a model where YopE, by its GAP activity inhibits Rho GTPases, and triggers Pyrin activation that is blocked by YopM. Future experiments will determine which Rho GTPases are involved in YopE-triggered caspase-1 cleavage via Pyrin. Priming appears necessary for *Y*. *pestis* ΔYopM to induce increased levels of IL-1β compared to the parental strain [1,4,6,13] (S3 Fig). This may be partly explained by the increased expression of Pyrin in the presence of TLR stimulation or killed *Y*. *pestis* (Fig 2F, S3 Fig), as baseline levels of Pyrin in macrophages may be low [22,23], although upregulation of Pyrin is delayed compared to IL-1β (Fig 2F). However, we cannot exclude the possibility that the regulation of other pathway members also plays a role. We also found that macrophages lacking the transcription factor C/EBPβ [24] were unable to produce IL-1β specifically in response to KIM5ΔYopM or KIM5ΔYopM/J (S4 Fig), and we note that transcription of Pyrin is controlled by C/EBPβ [25]. To test the hypothesis that the inhibition of the Pyrin pathway *in vivo* contributes to virulence, we infected WT, Pyrin KO, NLRP3 KO and IL-18 KO mice with the attenuated *Y*. *pestis* KIM1001 ΔYopM/J strain. For the fully virulent *Y*. *pestis* KIM1001 strain, the deletion of both these Yops is necessary for attenuation and increased IL-1b/IL-18 production *in vivo* [1]. We found that the attenuation of this *Y*. *pestis* strain lacking YopM, apparent in wild-type C57Bl/6 mice, could be completely reversed in Pyrin KO mice. These animals were highly susceptible to infection and all succumbed within a few days (Fig 2G), similar to caspase-1/11 deficient mice (Fig 2H). YopM is a strong inhibitor of Pyrin-mediated inflammasome activation, and we propose that the inhibition of this innate immunity pathway is a key feature of bacterially driven anti-host responses during plague, thus emphasizing the *in vivo* importance of our *in vitro* findings.

### YopK, but not YopM, keeps NLRP3 and NLRC4 activation by the Y. pestis needle/translocon in check

**Fig 3:**
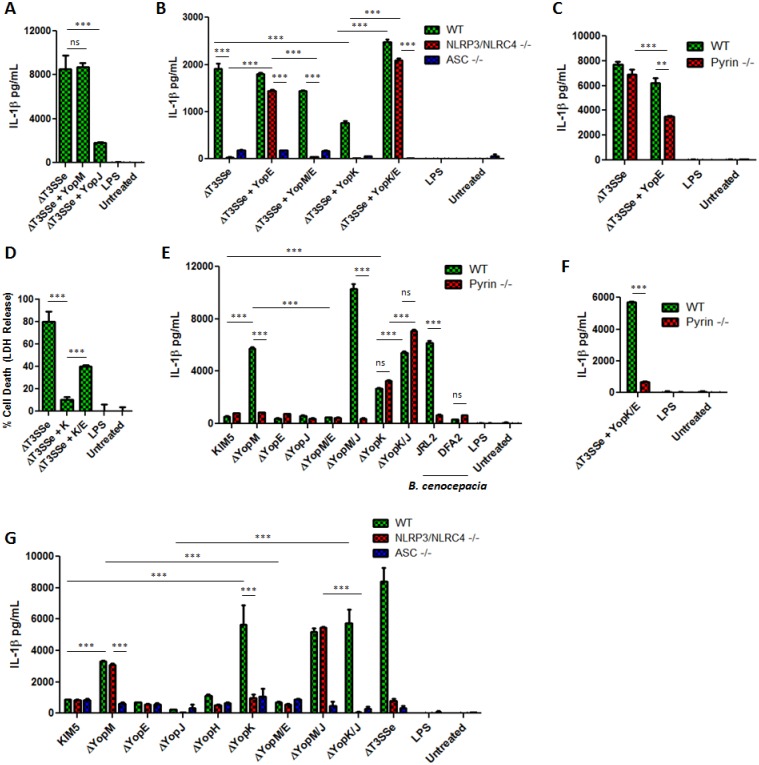
A-F,E,G) IL-1β was measured by ELISA in supernatants of LPS-primed BMDMs infected for 6 hours with indicated bacterial strains, or D) cell death was measured by LDH release at 6 hours p.i. (MOI 10). Figures are representative of three or more experiments. Shown is mean plus s.d. for triplicate wells. * p<0.05, **p<0.01, ***p<0.001.

As we were unsure whether effector YopK would inhibit Pyrin, we conducted a set of experiments probing involvement of YopK in the *Y*. *pestis*-triggered NLRP3/NLRC4 pathways compared to the Pyrin pathway. We confirmed the dependence of *Y*. *pestis* needle/translocon-mediated IL-1β secretion on caspase-1, NLRP3 and NLRC4 (Fig 1), suggested by studies in *Y*. *pseudotuberculosis* [3,11]. We observed a strong IL-1β induction in response to the ΔT3SSe lacking secreted effectors (Fig 1B). As reported previously, TNFα production is not significantly affected by the presence of the needle/translocon as compared to a strain (KIM6) lacking all T3SS components [1]. We next investigated whether reconstituting endogenous levels of YopM, YopK or YopJ on a ΔT3SSe background (inserted back onto the T3SS containing pCD1 plasmid by allelic exchange) would inhibit IL-1β release. The ΔT3SSe + YopJ strain demonstrated strong but incomplete inhibition of IL-1β, suggesting that inhibition of transcription may be a dominant response to YopJ in this case, and not the YopJ inflammasome activating ability. Importantly, ΔT3SSe + YopM had similar IL-1β induction as the ΔT3SSe (Fig 3A). This is consistent with the hypothesis that YopM inhibits the Pyrin inflammasome (Fig 2) but may not in this condition be a general inhibitor of caspase-1 [4]. We furthermore observed that Pyrin deficient macrophages released similar amounts of IL-1β in response to the needle/translocon expressing ΔT3SSe strain, emphasizing that the response to the presence of basic T3SS nano-machinery components requires NLRP3/NLRC4 but is independent of Pyrin. YopE may be an activator of the Pyrin pathway (Fig 2), and indeed, when expressing YopE in the ΔT3SSe strain the IL-1β release became less dependent upon NLRP3/NLRC4 (Fig 3B), and partly Pyrin dependent (Fig 3C). The NLRP3/NLRC4 dependence was fully restored when YopM was expressed in addition to YopE (Fig 3B). This suggests that YopM inhibits a YopE-triggered Pyrin pathway which is distinct from the needle/translocon triggered NLRP3/NLRC4 pathway.

Earlier data suggested that YopK may limit NLRP3 activation, either by preventing hypertranslocation of the pore-forming complex or regulating the injection of Yops [3,11]. Indeed, the addition of YopK reduces ΔT3SSe-triggered IL-1β effectively (Fig 3B), and also prevents ΔT3SSe induced cell death(Fig 3D). Interestingly, upon expression of the proposed Pyrin activator YopE in ΔT3SSe in addition to YopK, the IL-1β release became essentially independent of NLRP3/NLRC4 (Fig 3B) but fully dependent upon Pyrin (Fig 3E and 3F). When YopK is deleted from a KIM5 or KIM5 ΔYopJ background, we observed a sharp rise in IL-1β production and cell death compared to parental *Y*. *pestis* KIM5 both in primary macrophages and dendritic cells (Fig 3E, S5 Fig). This IL-1β production in response to infection with KIM5 ΔYopK was eliminated in NLRP3/NLRC4 deficient cells, but was independent of Pyrin (Fig 3F and 3G). We conclude that YopK is both necessary and sufficient to block inflammasome activation induced by the presence of the *Y*. *pestis* needle/translocon via NLRP3/NLRC4, but does not appear to block Pyrin activation induced by YopE. We cannot fully exclude the possibility that impact on translocation of other molecules by YopK and YopE [11,26,27,28,29] could be a factor in our observations. In contrast, IL-1β induced by the ΔYopM strain is fully dependent upon the presence of Pyrin, similarly to a *Burkholderia cenocepacia* strain (JRL2) that lacks a functional T3SS but expresses a Pyrin-activating T6SS (Fig 3E) [15,30,31,32]. For *B*. *cenocepacia*, the absence of the T6SS in the DFA2 strain strongly reduces the ability to trigger IL-1β release, in spite of a functional T3SS, underscoring how *Yersinia* and *Burkholderia* trigger Pyrin inflammasomes via different secretion systems. The picture that emerges is consistent with the hypothesis that YopM is an inhibitor of the Pyrin inflammasome triggered by YopE, whereas YopK inhibits NLRP3/NLRC4 activation triggered by the presence of the T3SS needle/translocon. This emphasizes a new complexity in the host inflammasome activation triggered by the *Y*. *pestis* T3SS.

### Pyrin is in a complex with YopM and RSK1, PKN1 kinases

**Fig 4:**
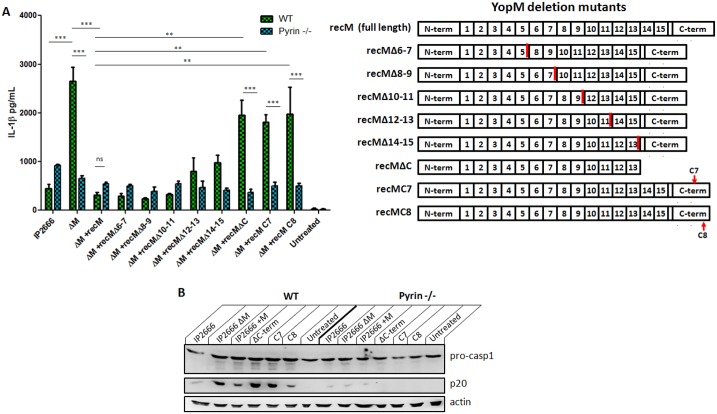
A) IL-1β was measured by ELISA in supernatants of LPS-primed BMDMs infected for 6 hours with *Y*. *pseudotuberculosis* IP2666, including strains expressing YopM with partial deletions (MOI 10). The numbers in the YopM protein refer to different leucine-rich repeat (LRR) domains of YopM, and C-term indicates the C-terminal end. RecM indicates reconstitution (rec) of IP2666 ΔYopM with variants of YopM, as shown in the figure. C7 and C8 are two different triple alanine substitutions near the C-terminus of YopM [33]. B) Total protein from LPS-primed BMDMs infected with indicated strains (combined cell lysate and supernatant) was separated by SDS-PAGE and analyzed by Western Blot for IL-1β and caspase-1. Figures are representative of two independent experiments. Shown is mean plus s.d. for triplicate wells. * p<0.05, **p<0.01, ***p<0.001.

*Y*. *pestis* is a close relative to *Y*. *pseudotuberculosis*, a human enteric pathogen. We confirmed that *Y*. *pseudotuberculosis* also induced IL-1β via Pyrin in the absence of YopM (Fig 4A). YopM has been proposed to interact with kinases of the PKN and RSK families. YopM consists of multiple leucine-rich repeat (LRR) domains, and several deletions of these LRR domains have been generated [33]. Using a *Y pseudotuberculosis* IP2666 strain reconstituted (rec) with YopM mutants on a ΔYopM background [33], we determined that the C-terminus of YopM is needed to inhibit Pyrin-mediated IL-1β release and caspase-1 processing (Fig 4A and 4B). In fact, alanine substitutions of only the last three C-terminal YopM amino acids in the recM C8 strain essentially prevented the ability of YopM to block IL-1β production (Fig 4A). This is also the region of YopM necessary for interaction with RSK1 kinase, [34], and these domains partly overlap with YopM regions interacting with PKN1 [34].

**Fig 5:**
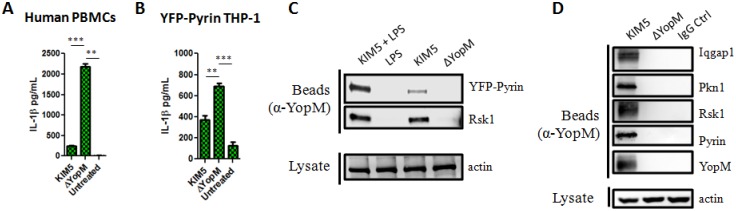
YopM maintains an inhibitory phenotype in human PBMCs, and in a human THP-1 cell line overexpressing YFP-Pyrin. Co-IP pulldown in these cells as well as mouse BMDMs indicate YopM interaction with Pyrin, Rsk1, Pkn1, and Iqgap1. A) PBMCs were isolated from healthy human donor blood and infected at MOI 10 with indicated *Y*. *pestis* strains without priming. At 6 hours p.i. supernatant was collected for IL-1β detection by ELISA. B) Cultured YFP-Pyrin THP-1 cells were differentiated with 100nM Vitamin D3 for 48–72 hours, and infected with indicated *Y*. *pestis* strains at MOI 10. Shown is IL-1β assayed from supernatants by ELISA at 6 hrs p.i. Figures are representative of three or more experiments. Shown is mean plus s.d. for triplicate wells. * p<0.05, **p<0.01, ***p<0.001. C-D) Shown are Western blot results of co-IP with anti-YopM using C) Vitamin D3-differentiated, unprimed YFP-Pyrin cells or D) LPS-primed BMDMs after infection with the indicated strains at MOI 10 for 3 hours. Bead-bound protein and lysates were separated by SDS-PAGE and analyzed by Western Blot for the proteins indicated.

As human and mouse pyrin exhibit some differences in structure, we wanted to confirm whether YopM is capable of inhibiting IL-1β induction in human cells. To do this, we isolated peripheral blood mononuclear cells (PBMCs) and infected them with *Y*. *pestis*. For this experiment, cells were not primed as PBMCs are known to produce IL-1β in response to TLR stimuli alone in an ERK-dependent manner [35]. We confirmed that YopM also contributes to inhibition of IL-1β secretion in human PBMCs (Fig 5A).

We next tested the effect of YopM in a monocytic human THP-1 cell line expressing YFP-Pyrin, this line was chosen as matured THP-1 cells have minimal endogenous levels of Pyrin. We found an IL-1β secretion pattern generally comparable to human PBMCs, where YopM is also capable of inhibiting IL-1β production (Fig 5B). We used the THP-1 YFP-Pyrin cells as a tool for biochemical analysis of how YopM or Rsk1 interacts with Pyrin [33,34,36]. Rsk1, and recently the cytoskeletal scaffolding protein Iqgap1, have been suggested to be important for caspase-1 inhibition by YopM [13]. Pull-down assays in THP-1 YFP-Pyrin cells and mouse BMDMs using anti-YopM antibody showed that YopM interacts with a complex containing Rsk1, PKN1, Pyrin and Iqgap1 (Fig 5C and 5D). As we also detected Pyrin in the bound fraction, it may directly or indirectly (via the kinases) interact with YopM.

**Fig 6:**
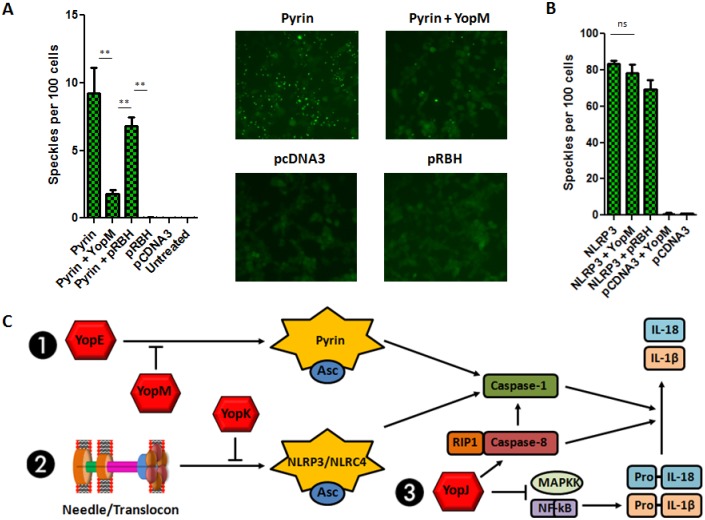
YopM prevents the formation of Pyrin-dependent but not NLRP3-dependent Asc complexes. A) HEK293T cells stably expressing Asc-YFP were transfected with pCDNA3-Pyrin, pRBH-YopM, or both constructs together. B) pCDNA3-NLRP3 and respective empty vectors were used as positive and negative controls. Asc speckles were visualized, quantified, and normalized to cell number. Figures are representative of three or more experiments. Shown is mean plus s.d. for triplicate fields quantified. * p<0.05, **p<0.01, ***p<0.001. C) Proposed model integrating the major interactions of the *Y*. *pestis* T3SS with inflammasome pathways.

Next, we tested the ability of YopM alone to inhibit Pyrin activation. HEK293T cells stably expressing Asc-YFP were transiently transfected with plasmids encoding Pyrin, YopM, or both constructs together. We observed significantly increased Asc complex (speckle) formation upon transfection of Pyrin, visualized as numerous fluorescent puncta, indicating inflammasome assembly following Pyrin over-expression (Fig 6A). This Asc speckling was significantly reduced upon co-transfection of YopM. The expression of NLRP3 also triggered the formation of Asc-speckles, but was not affected by the co-expression of YopM (Fig 6B), suggesting YopM specificity for inhibiting the Pyrin pathway.

## Discussion

Taken together, our data show complex interactions of a bacterial T3SS with host inflammasome signaling pathways. We have identified several pathways triggered by *Y*. *pestis* T3SS, as both Pyrin, caspase-8 and NLRP3/NLRC4 appear directly and distinctly involved in regulation of caspase-1 cleavage and IL-1β release. Two secreted T3SS effectors, YopK and YopM, appear to be specific inhibitors of the NLRP3/NLRC4 activation induced by the presence of the T3SS needle/translocon, and the YopE-induced Pyrin activation, respectively. Inflammasome activation by YopE may represent a process where the host innate immune system acquired the ability to sense damaging microbial interference and inhibition of a specific pathway (RhoA signaling). Our current model of how the *Y*. *pestis* T3SS components intersect with specific inflammasome pathways is shown in Fig 6C. Both YopK and YopM are participating in maximal suppression of innate immunity following *Y*. *pestis* infection, and are central components of the arsenal that this highly virulent pathogen allocates to interference with key anti-bacterial immune responses. Our findings highlight the remarkable sophistication that *Yersinia* displays when interfacing with inflammasome signals.

Previously, covalent modifications of RhoA by bacteria were proposed to trigger Pyrin activation [17]. Our data suggest an additional way that bacteria initiate Pyrin-dependent mechanisms, as we here propose that the GAP effector YopE activate the cascade leading to Pyrin activation. Thus, changes in the Rho GTPase phosphorylation status could trigger the cascade leading to Pyrin-mediated IL-1β release and cell death. It would be interesting to see whether other bacterial GAPs also harbor this ability, such as *Salmonella enterica* serovar Typhimurium effector SptP [37,38], although this effector has not been suggested to target RhoA. We also cannot exclude the possibility that other Rho family modifying effectors, such as *Y*. *pestis* YopT which cleaves Rho GTPases, also can contribute, although in *Y*. *pestis*, the deletion of YopE fully abolishes the additional IL-1β triggered by the ΔYopM strain. We can also extend the discussion of possible Pyrin pathway modifiers to consider bacterial Rho GTPase exchange factors (GEFs), activating Rho family proteins, as players in the system [37]. Indeed, the symmetry axis between YopE and YopM that simultaneously activates and inhibits signaling mediated by RhoGTPases also has parallels in other pathogens. Of note, *S*. Typhimurium expresses both SptP (with GAP activity towards Rac1) and SopE-SopE2 (with GEF activity), highlighting the complex regulation of cellular activation by bacterial T3SS effectors. For both the YopE/YopM and SptP/SopE-SopE2 sets, the inhibiting effectors may be necessary only when the activating effector is present. This type of observation may also open up for hypotheses about sequential evolution of specific effector proteins. The requirement for the inhibitor YopM only in the presence of the activator YopE suggests that the latter may have been acquired first, and hence hints at the evolutionary steps that produced the extant system. More generally, it reveals interplay between the host and pathogen that may in part drive the selection for increasing complexity of Type III secretion systems: addition of a new effector is favored because it counters the host responses driven by the one most recently acquired.

Other aspects that can impact Pyrin signaling include mutations in Pyrin itself [16,39], mutations in mouse WDR proteins impacting actin depolymerization [40], and alterations of the mevalonate pathway [41,42] which can activate Pyrin inflammasomes. This implies that a number of different ways to trigger Pyrin may exist, but this also widens the range of potential microbial or pathological impact of this pathway. Also, historical plague pandemics in Europe started in the same area where FMF is most prevalent, in the Mediterranean basin. Although difficult to verify, it is possible that some FMF-related mutations caused altered susceptibility to infection, and this could have contributed to modified host responses to bacteria [43].

Several regulators of the Pyrin inflammasome pathway have recently been proposed, including 14-3-3 proteins [39] and PKN1/2 kinases [41]. Interestingly, PKN kinases also bind to YopM, opening up for future studies of direct roles of these interactions on Pyrin activation. Here we also show that RSK1 kinase binds a complex of YopM, PKN1 and Pyrin. One attractive model involves phosphorylation of Pyrin [39,41] by YopM-interacting kinases and stabilization of the inactivated and phosphorylated Pyrin by 14-3-3 proteins. The complex may also involve the scaffold protein Iqgap1 [13]. This model may also include one or more phosphatases that will de-phosphorylate Pyrin under stimulating conditions, although such a phosphatase has not yet been identified.

Very recently, a paper was published showing that YopM from *Y*. *pseudotuberculosis* inhibits the Pyrin inflammasome triggered by YopE [44]. Both that study and ours demonstrate that attenuated YopM or YopM/J mutant strains re-gain virulence *in vivo* in the absence of Pyrin. Similar to the mechanism what we suggest with *Y*. *pestis*, the report indicated that *Y*. *pseudotuberculosis* YopM interacts with kinases PKN1/2 and RSK1 in a complex with Pyrin, but also that the kinases phosphorylate Pyrin which in turn is stabilized in a phosphorylated inactive state by 14-3-3 proteins [44]. One difference between the two papers is that Chung et al also suggests that YopT, via its protease activity towards RhoA, also can trigger Pyrin activation. Although we have not studied YopT in detail, it appears from our experiments that on a *Y*. *pestis* deltaYopM background, YopE fully accounts for the Pyrin activating ability and that a dual deletion of YopM and YopE brings IL-1β release and caspase-1 cleavage down to the level of the parental strain. Some of these differences may be explained by differences in YopE GAP activity between *Y*. *pestis* and *Y*. *pseudotuberculosis*, or by differences in experimental conditions.

Another possibility is higher basal inflammasome activation mediated by *Y*. *pestis* KIM YopJ via caspase-8, which may also explain why the parental *Y*. *pestis* strain has a fairly strong IL-1β release and caspase-1 cleavage (Figs 1 and 2) compared to the low (Fig 4) or absent [44] caspase-1 cleavage or IL-1β release observed with *Y*. *psedotuberculosis*. This difference may very well be explained by the markedly higher enzymatic activity of *Y*. *pestis* KIM YopJ compared to *Y*. *pseudotuberculosis* YopJ, as previously proposed [8]. Nevertheless, despite small differences mentioned above in between these two reports, we can summarize that *Y*. *psedotuberculosis* and *Y*. *pestis* YopM proteins both engage in similar inhibitory activity towards Pyrin-dependent inflammasome activation, and are central in the strategy of both pathogens to inhibit innate immune responses in their favor.

In conclusion, we have identified *Y*. *pestis* YopM as a microbial inhibitor of the Pyrin inflammasome pathway. Detailed knowledge about the mechanisms that the T3SS effector YopM influences may open up for the development of novel treatments in Pyrin-mediated diseases.

## Methods

### Mice

Most mouse strains used in this study were described previously [2,24]. Pyrin (Mefv) -/- mice lacking exons 1–4 mice were generated at Genentech from gene- targeted C57BL/6N C2 ES cells. Alternatively, Pyrin -/- mice provided by Jackson Laboratories were utilized. BMDMs were differentiated from bone marrow harvested from the femurs of 6–20 week old mice. Mice were injected s.c. with 160 CFU KIM1001ΔYopM/J and monitored for survival.

### Bacterial strains and growth conditions

The fully virulent KIM1001 strain of *Y*. *pestis*, the pgm-deficient but pCD1+ strain KIM5 (containg the full T3SS) as well as its mutant derivatives (ΔYopJ, ΔYopM, ΔYopM/J, ΔT3SSe and the KIM6 strain entirely lacking the T3SS-encoding plasmid pCD1), and the *Y*. *pseudotuberculosis* IP2666ΔYopM as well as IP2666ΔYopM+recM (reconstituted with YopM variants) mutant strains were previously described [1,5,7,45,46]. The strains were generated by in-frame deletions and allelic exchange. The T3SS secreted effector deficient strain (ΔT3SSe) was constructed by making sequential in-frame deletions on KIM5 [1]. This strain lacks Yops M, E, J, H, T, K and YpkA, but expresses pore-forming translocon components Yops B, D and the machinery necessary to assemble a T3SS needle/rod. The full-length genes of *yopK*, *yopM*, or *yopE* were restored onto the ΔT3SSe background on the pCD1 plasmid. *Y*. *pestis* strains were grown in tryptose-beef extract broth (2xYT for *Y*. *pseudotuberculosis* strains) with 2.5mM CaCl_2_. Bacteria were added to cells at MOI 10.

### Cell stimulations

Bone marrow derived macrophages (BMDMs) from mice in our facility were differentiated in RPMI 1640 supplemented with 10% fetal calf serum (FCS), 25mM HEPES, 10ug/mL ciprofloxacin, and 10% L929 conditioned medium containing M-CSF for 5 days [1]. Cells were primed with 100 ng/mL *E*. *coli* O111:B4 LPS for 5 hours or allowed to rest in antibiotic-free RPMI with 10% FCS and 25mM HEPES without antibiotic before addition of bacteria at an MOI of 10. IL-1β/IL-18 Elisa, LDH cell death assays and immunoprecipitation and western blots were performed as described in supplemental material. HEK293 Asc-YFP cells were provided by K. Fitzgerald and THP-1-Pyrin-YFP cells from M. Wewers and M. Gavrilin [32]. Human PBMC were obtained from donor whole blood (harvested in our lab at UMass) using Lymphoprep density gradient (Axis-Shield) and stimulated in RPMI 1640 supplemented with 10% FCS and 25mM HEPES. TcdA and TcdB were from List Biological Labs.

### Statistical analysis

*In vitro* assays were analyzed by two-way ANOVA followed by Bonferroni post-test. Differences in mouse survival were analyzed by Kaplan-Meyer analysis and logrank test. Values where p < 0.05 were considered significant.

### Ethics statement

All animal studies were performed in compliance with the federal regulations set forth in the Animal Welfare Act (AWA), the recommendations in the Guide for the Care and Use of Laboratory Animals of the National Institutes of Health, and the guidelines of the UMass Medical School Institutional Animal Use and Care Committee. All protocols used in this study were approved by the Institutional Animal Care and Use Committee at the UMass Medical School (protocols A-2332 and A-2339). Human PBMC were obtained from healthy volunteer donor whole blood collected in our lab at UMass Medical School after donor review of information fact sheet and oral consent. The research enrolled only adult subjects, and all provided informed consent. In accordance with U.S. Code of Federal Regulations 45 CFR 46.117(c)(2), the UMass Medical School Institutional Review Board approved oral consent and waived written documentation of consent as the research and phlebotomy presented no more than minimal risk of harm to subjects and involved no procedures for which written consent is normally required outside of the research context. The consent of each individual was recorded as demographic information according to requirements by the National Institutes of Health. All human subject work was conducted in accordance with the guidelines given by the Institutional Review Board at UMass Medical School, and approved by the same Board (protocol H-11183).

More detailed methods are found in S1 Supporting information.

## Supporting Information

S1 Supporting Information Supporting Methods. (PDF)

S1 Table Primers and oligos used for generation of bacterial strains. (DOCX)

S2 Table Primers used for RT-PCR. (DOCX)

S1 Fig The IL-1β pathway inhibited by YopM is not dependent on NLRP12, RIP3, or caspase-8. LPS-primed BMDMs were infected with indicated strains of *Y*. *pestis* at MOI 10 for 6 hours, and supernatant IL-1β was assayed by ELISA in A) WT, RIP3/Caspase-8 -/- and B) WT, NLRP12 -/-, RIP3 -/-, BMDMs. Decrease of IL-1 release in the absence of caspase-8 cannot be explained by reduced caspase-1 cleavage (Fig 1), but rather reflects reduced transcriptional activity. C) Total protein from LPS-primed RIP3 -/- BMDMs infected with indicated strains (combined cell lysate and supernatant) was separated by SDS-PAGE and analyzed by Western Blot for caspase-1.
(TIF)

S2 Fig The inflammasome response to the RhoA-inhibiting *Clostridium difficile* toxins A and B is Pyrin-dependent. LPS-primed (100 ng/ml) BMDMs of indicated genotypes (WT C57Bl/6 or KO) were treated with 0.2uM TcdA, 0.2uM TcdB, or 5mM ATP. A) supernatant IL-1β was assayed by ELISA and B) cell death was assayed by LDH assay.
(TIF)

S3 Fig Robust activation of the Pyrin-dependent pathway requires priming. Priming can be achieved with LPS or heat-killed bacteria expressing either hexa- or tetra-acylated LPS. The suppressive action of YopJ appears to contribute to the need for priming. A) 100ng/mL LPS or 1x10^8^ CFU equivalents of heat-killed KIM5 were added to BMDMs either 5 hours before infection, or simultaneously with live KIM5 or ΔYopM at MOI 10. Supernatant from 6 hours p.i. was assayed for IL-1β by ELISA. B) Priming can be achieved with heat-killed *Y*. *pestis* regardless of whether it is grown at 26°C or 37°C, despite expression of tetra-acylated LPS with low stimulatory ability. C) Unprimed BMDMs were infected with indicated strains of *Y*. *pestis* (temperature-shifted) at MOI 10 for 6 hours, and supernatant IL-1β was assayed by ELISA. It is also worth noting that without priming, KIM5ΔYopM produces IL-1β comparable to parental KIM5, whereas KIM5ΔYopM/J triggers significantly elevated levels of IL-1β (S3 Fig). It is possible that YopJ suppresses priming that occurs during the course of the 6-hour infection, either by inhibiting NF-κB- or MAPK mediated gene expression, or by inducing apoptosis before sufficient priming can occur. This is further suggested by the fact that LPS-priming is not required to elicit a strong IL-1β response with KIM5ΔYopM/J, unlike KIM5ΔYopM where YopJ is present.
(TIF)

S4 Fig C/EBPβ is specifically required for activation of the Pyrin-dependent IL-1β pathway which YopM inhibits. LPS-primed BMDMs were infected with indicated strains of *Y*. *pestis* at MOI 10 for 6 hours, and supernatant IL-1β was assayed by ELISA.
(TIF)

S5 Fig YopK is required to inhibit needle/translocon induced IL-1β and cell death in dendritic cells in addition to macrophages. LPS-primed BMDCs were infected with indicated strains of *Y*. *pestis* at MOI 10 for 6 hours; A) supernatant IL-1β was assayed by ELISA, and B) cell death was measured by LDH release.
(TIF)
