# Supplementary material for: The Yersinia pestis Effector YopM Inhibits Pyrin Inflammasome Activation
Source: PLoS Pathog. 2016 Dec 2;12(12):e1006035. doi: 10.1371/journal.ppat.1006035 (PMC5135138; doi:10.1371/journal.ppat.1006035)
Supplement: S1 Supporting Information — (PDF) [file ppat.1006035.s001.pdf]

Supporting Information

S1 Supporting Methods

Mice. All experiments involving mice were approved by the Institutional Animal Care and Use Committee. Most mouse strains used in this study were described previously(1) and bred in-house. Pyrin (Mefv) -/- mice lacking exons 1-4 were generated at Genentech from gene- targeted C57BL/6N C2 ES cells. PCR primers (5'-CAGGCTACAGGGAGACAAGAA; 5'-TCCTACCATTGCCACTGAGAG and 5'-CAAAGGGAGCACAGACACTTC) were used to genotype Pyrin -/- mice, with a 184-bp wild-type DNA fragment and a 265-bp mutant DNA fragment produced. Alternatively, Pyrin -/- mice provided by Jackson Labs were utilized. C/EBPβ -/- mice were generated by Dr. Johnson at the National Cancer Institute(2). BMDMs and BMDCs were differentiated from bone marrow harvested from the femurs of 6-20 week old mice. Subcutaneous (s.c.) inoculations were conducted by injecting mice with 160 CFU Y. pestis KIM1001 ΔYopM/ΔYopJ in 50μL of PBS in the nape of the neck; mice were then monitored for survival for up to 30 days.

Bacterial Strains and Growth Conditions. The fully virulent KIM1001 strain of *Y. pestis,* the attenuated KIM5 (Δ*pgm*) BSL2 strain, and KIM5ΔYopJ were previously described(3-5). The ΔYopM, and ΔYopM/J strains were generated both on the KIM5 and KIM1001 background as follows: An in-frame deletion removing amino acids 3 through 408 of 410 of the *yopM* gene was created via allelic exchange. PCR products made with primer sets yopM-A, yopM-B and yopM-C, yopM-D, respectively, were used to make a fused product by overlap PCR using primers A and D(6). This product was cloned in the allelic exchange vector pRE107(7) in *E.coli* K12 strain β2155, a diaminopimelic acid auxotroph, and transferred to *Y. pestis* KIM1001 by conjugation. KIM1001 recombinants were selected on TB medium containing 100μg/ml ampicillin but no diaminopimelic acid. Following counter selection with 5% sucrose, deletion mutants were identified by PCR. The same procedure was followed to construct an in-frame deletion mutant of *yopJ*, *yopE,* and *yopK* in KIM1001, as well as *yopJ,* and *yopE* in KIM1001 Δ*yopM* and KIM1001 Δ*yopK* using the respective gene specific A, B, C, and D primers shown in Table S1. Attenuated Δ*pgm* derivatives of each strain, bearing the designation KIM5 to indicate their altered chromosomal genotype, were derived from their respective KIM1001 parents by selection for loss of pigmentation on HIB Congo Red agar at 26°C. Loss of the pigmentation region (Δ*pgm*)/iron acquisition was confirmed by PCR with primers pgm-F, pgm-R; psn-F, psn-R; and hmsH-F, hmsH-R. Expression of YopM and YopJ was confirmed by RT-PCR.

A Type III secretion effector deficient strain (ΔT3SSe) was constructed by making sequential in-frame deletions, as described above, of *yopM* (amino acids 3-408 of 410), *yopE* (amino acids 40-197 of 220), *yopH* (amino acids of 3-467 of 469), *ypkA* (amino acids of 3-731 of 733), *yopJ* (amino acids 4-288 of 289), *yopK* (amino acids 4-181 of 183), and *yopT* (amino acids of 3-320 of 323) using the respective gene specific A, B, C, and D primers shown in Table S1. The deletions were made in *Y. pestis* KIM 1001 and a KIM5 derivative was generated as described above. This strain lacks Yops M/E/J/H/T/K and YpkA, but expresses Yops B/D and the machinery necessary to assemble a T3SS needle with a functional pore-forming translocon complex. To examine the contribution of individual Yops, the full-length genes of *yopK*, *yopM*, or *yopE* were restored to the ΔT3SSe background on the pCD1 plasmid. PCR products made with each respective yop's primer A and D (see Table S1), using wild-type KIM5 template, were cloned into pRE107 and allelic exchange carried out as described above. Strains expressing pairs of Yops (only YopM and YopE, and only YopK and YopE) were constructed by restoring the full-length *yopE* gene to the ΔT3SSe *yopM*+ or ΔT3SSe *yopK*+ backgrounds.

All *Y. pestis* strains were grown using TB media supplemented with 2.5mM $CaCl_2$. KIM1001 and derivative strains were plated on agar incubated at 37°C overnight and passed once before preparing inoculum for injection. Strains on the KIM5 background were plated overnight from frozen glycerol stocks and then grown at 26°C in liquid broth overnight; on the day of infection cultures were diluted 1:20 and grown for 2 hours at 26°C followed by a shift to 37°C for 2 hours. This transition is important to up-regulate expression of the T3SS and allowing for the presence of some TLR4-activating LPS, while minimizing expression of F1 protein capsule(8), which interferes with cell-based assays. Bacteria were

then washed three times in RPMI (pre-warmed to 37°C), quantified by OD600, and added to cells at a multiplicity of infection (MOI) of 10 bacteria per cell in a 10uL volume within 1 hour of preparation.

Y. pseudotuberculosis IP2666ΔYopM and IP2666ΔYopM+recM mutant strains were a provided by Dr. Joan Mecsas and have been previously described(9). *Y. pseudotuberculosis* strains were grown identically to the KIM5-background strains described above, except 2xYT media was used instead of TB (also supplemented with 2.5mM $CaCl_2$).

Heat-killed KIM5 for priming was prepared by growing KIM5 in liquid broth from freshly streaked plates overnight either at 26°C or 37°C. Bacteria were diluted 1:20, and grown for an additional three hours at the same temperature. Bacteria were then washed three times in endotoxin-free PBS, quantified by OD600, and incubated at 65°C for 1 hour to kill the bacteria. An aliquot from each condition was plated to confirm 100% death, and the rest was frozen. For priming in subsequent experiments, an equivalent of MOI 10 of either prep was used. *Burkholderia cenocepacia* is a Gram-negative bacteria observed in lungs of a number of patients with cystic fibrosis, is often problematic to treat due to high resistance to antibiotics, and its presence prevents eligibility for lung transplantation. *B. cenocepacia* strains JRL2 (expressing T6SS but without a functional T3SS due to inactivation of the bcsV gene) and DFA2 (expressing T3SS but without a functional T6SS, due to a deletion in the icmF gene)(10), both derived from a K56-2 strain, were provided by Drs. A. Amer (Ohio State Univ.) and M. Valvano (Queen's University Belfast).

Cells and Stimulations. Bone marrow derived macrophages (BMDMs) were obtained from bone marrows of mice in our facility at UMass and differentiated in RPMI 1640 supplemented with 10% fetal calf serum (FCS), 25mM HEPES, 10ug/mL ciprofloxacin, and 10% L929 conditioned medium containing M-CSF for 5 days. Bone marrow derived dendritic cells (BMDCs) were differentiated in R10 medium consisting of RPMI 1640, 10% FCS, 20mM HEPES, 2mM L-glutamine, 50μM β-mercaptoethanol, 100U/mL penicillin, 100μg/mL streptomycin, and 20ng/mL recombinant murine GM-CSF (Peprotech) for 9 days. BMDMs or BMDCs were harvested and seeded at a density of 100,000 cells per well in a 96-well plate format overnight and stimulated the following day. HEK293T cells stably expressing Asc-YFP (provided by Dr. K. Fitzgerald, UMass) were maintained in DMEM supplemented with 10% fetal calf serum (FCS), 25mM HEPES, and 10ug/mL ciprofloxacin. THP-1 cells stably expressing YFP-Pyrin and siPyrin(11), generated by Drs. Wewers and Gavrilin, were maintained in RPMI 1640 supplemented with 10% fetal calf serum (FCS), 25mM HEPES, and 10ug/mL ciprofloxacin. For stimulations, THP-1 cells were differentiated for 48 hours with 100nM Vitamin D3, harvested, and seeded for same day stimulation at either 100,000 cells in 96-well plate format, or at $1x10^7$ cells per 10cm dish for immunoprecipitation experiments. Human PBMCs were isolated from healthy volunteer donor whole blood, harvested at UMass, using Lymphoprep density gradient (#07851, StemCell, Axis-Shield), washed once, and resuspended in RPMI 1640 supplemented with 10% fetal calf serum (FCS), 25mM HEPES. PBMCs were seeded at a density of 100,000 cells per well in a 96-well plate format and immediately used for infection.

BMDM were primed with 100 ng/mL LPS for 5 hours or allowed to rest in antibiotic-free RPMI with 10% FCS and 25mM HEPES without antibiotic before addition of bacteria at an MOI of 10. Endpoints were as follows: 6 hours p.i. for LDH assay (CytoTox 96, Promega #G1780), 6 hours for harvesting of supernatant and/or cells for analysis of cytokines by ELISA (R&D) or caspase-1 activation by SDS-PAGE and Western Blot (anti-caspase-1 #AG-20B-0042-C100, Adipogen; anti-IL-1β #DY401 840135, R&D), and 3 hours for immunoprecipitations. For time points exceeding 3 hours, 50ug/mL gentamicin was added at 3 hours p.i. Each graph represents results of two or more independent cell stimulations on separate days.

Immunoprecipitations and western blots. Cells were infected at MOI 10 as described above, and harvested either at 3 hours or 6 hours p.i., with 50ug/mL gentamicin added at 3 hours. Cells were washed once and lysed in co-IP buffer for 15 minutes (1% Triton X-100, 150mM NaCl, 5mM KCl, 2mM MgCl2, 1mM EDTA, 25mM Tris-HCl, pH 7.4) with protease and phosphatase inhibitor (#04693116001 and #04906845001, Roche). Lysate was cleared by centrifugation and a fraction was saved as loading input control. The remaining lysate was cleared once with protein G agarose beads (Thermo #20398) to remove non-specific protein binding, followed by incubation with fresh beads and pull-down antibody against

YopM (#NR-801, BEI Resources) for 2 hours at 4°C. Beads were then washed five times in co-IP buffer, and bound proteins were eluted by direct addition of SDS loading buffer buffer with 1mM DTT. Beads and saved lysate were analyzed by SDS-PAGE and analyzed by Western Blot (anti-Rsk1 #sc-231, Santa Cruz; anti-Pyrin polyclonal antibody(11).

RT-PCR. RNA was extracted using the QIAGEN RNEasy kit, followed by RT-PCR with the SYBR Green DNA probe (BioRad). C(t) values were normalized to GAPDH internal controls and the means were normalized to the unstimulated negative control group. Primers used for RT-PCR are listed in Table S2.

Mammalian expression vectors. The pCDNA3-mPyrin plasmid was generated by Dr. Alnemri(12). pcDNA3-mNLRP3 was from K. Fitzgerald (UMass). pRBH-YopM was constructed as follows: endogenous YopM was amplified out of pCD1 by direct PCR on KIM5 bacteria using primers YopM-F and YopM-R (see Table S2). Self-complementary oligos (Oligo 5.1 and Oligo 5.2) coding a his-tag sequence along with part of the YopM N-terminus were designed and annealed to each other, resulting in 5' and 3' overhangs compatible for subsequent ligation. The YopM PCR product and pRBH vector were digested with BsrFI/XhoI and BamHI/XhoI respectively, gel-purified, and ligated together with the annealed Oligos 5.1 and 5.2. This resulted in YopM containing a His6 sequence immediately following the first methionine, inserted downstream of the CMV promoter and upstream of an mCherry reporter gene. The resulting pRBH-YopM construct was then cloned and maintained in TOP10 cells (Invitrogen).

Transfections. HEK293T-Asc-YFP cells were seeded at $1x10^6$ cells per well in 24-well format, and transfected with pcDNA3-Pyrin, pcDNA3-NLRP3, pRBH-YopM, or empty control plasmids using Lipofectamine 2000 (Thermo #11668030). 250ng of each plasmid was transfected together with 250ng of either pRBH-YopM or empty control vector for a constant total of 500ng per well and no more than 250ng of any one plasmid. After 24 hours cells were stained with Hoechst 33342 and visualized using an epifluorescent microscope. Asc complex formation puncta (speckles) were quantified and normalized to cell number in three separate fields per sample, using a standardized batch algorithm in Adobe Photoshop CS4.

Statistical Analysis. *In vitro* assays were analyzed by two-way ANOVA followed by Bonferroni post-test. Differences in mouse survival were analyzed by Kaplan-Meyer analysis and logrank test. Values where $p < 0.05$ were considered significant.

References

1. Vladimer GI, *et al.* (2012) The NLRP12 inflammasome recognizes Yersinia pestis. *Immunity* 37(1):96-107.
2. Sterneck E, Tessarollo L, & Johnson PF (1997) An essential role for C/EBPbeta in female reproduction. *Genes Dev* 11(17):2153-2162.
3. Montminy SW, *et al.* (2006) Virulence factors of Yersinia pestis are overcome by a strong lipopolysaccharide response. *Nat Immunol* 7(10):1066-1073.
4. Sodeinde OA, *et al.* (1992) A surface protease and the invasive character of plague. *Science* 258(5084):1004-1007.
5. Weng D, *et al.* (2014) Caspase-8 and RIP kinases regulate bacteria-induced innate immune responses and cell death. *Proc Natl Acad Sci U S A* 111(20):7391-7396.
6. Horton RM, Hunt HD, Ho SN, Pullen JK, & Pease LR (1989) Engineering hybrid genes without the use of restriction enzymes: gene splicing by overlap extension. *Gene* 77(1):61-68 .
7. Edwards RA, Keller LH, & Schifferli DM (1998) Improved allelic exchange vectors and their use to analyze 987P fimbria gene expression. *Gene* 207(2):149-157.
8. Lenz JD, *et al.* (2011) Expression during host infection and localization of Yersinia pestis autotransporter proteins. *J Bacteriol* 193(21):5936-5949.
9. McCoy MW, Marre ML, Lesser CF, & Mecsas J (2010) The C-terminal tail of Yersinia pseudotuberculosis YopM is critical for interacting with RSK1 and for virulence. *Infect Immun* 78(6):2584-2598\.

10. Aubert D, MacDonald DK, & Valvano MA (2010) BcsKC is an essential protein for the type VI secretion system activity in Burkholderia cenocepacia that forms an outer membrane complex with BcsLB. *J Biol Chem* 285(46):35988-35998.
11. Gavrilin MA, *et al.* (2009) Pyrin critical to macrophage IL-1beta response to Francisella challenge. *J Immunol* 182(12):7982-7989 .
12. Yu JW, *et al.* (2007) Pyrin activates the ASC pyroptosome in response to engagement by autoinflammatory PSTPIP1 mutants. *Mol Cell* 28(2):214-227 .
