# Supplementary material for: The Yersinia pestis Effector YopM Inhibits Pyrin Inflammasome Activation
Source: PLoS Pathog. 2016 Dec 2;12(12):e1006035. doi: 10.1371/journal.ppat.1006035 (PMC5135138; doi:10.1371/journal.ppat.1006035)
Supplement: S1 Table — (DOCX) [file ppat.1006035.s002.docx]

**Table S1**. Primers and oligos used for generation of bacterial strains

| **Primer/Oligo Name** | **Sequence 5’ to 3’** |
| --- | --- |
| yopM-A | ATAGAGCTCTTCAAAAGGGGTACTGGATAC |
| yopM-B | GAACATATTGAATGCCTTTCT |
| yopM-C | AGAAAGGCATTCAATATGTTCGAGTAGTACGCAAGAGCGTTC |
| yopM-D | GGGTCTAGATTTACCAATTTTTTGATGGGG |
| yopJ-A | ATAGAGCTCCACTACTGATTCAACTTGGACG |
| yopJ-B | ACGGCAAATGCAGAGCAGTCCGATCATTTATTTATCCTTATTCA |
| yopJ-C | CTGCTCTGCATTTGCCGTTAATGTATTTTGGAAATCTTGCT |
| yopJ-D | GGGTCTAGACTGATGTCGTTTATTTCTGGGTAT |
| yopE-A | ATAGAGCTCAGCATTACACACTCCACAGTTGGGT |
| yopE-B | ACGCAGGCAGCAAATGAGATCAAA |
| yopE-C | CTCATTTGCTGCCTGCGTATATTGATCACTTGTTTG |
| yopE-D | ATATCTAGATATCCAGGCTGTTCAATGGTTGTCGAT |
| yopK-A | GGGGAGCTCTGTTAGCCATTATTTTGCTATAC |
| yopK-B | ACGGCAAATGCAGAGCAGAATAAACATAGTTACTACTCCCAAA |
| yopK-C | CTGCTCTGCATTTGCCGTGGATGAAGCTATATTAAAGAGTT |
| yopK-D | ATATCTAGACATTTAAAACAGGGCATGG |
| Pgm-F | CCGCAACAACATCATCCGTATTCA |
| Pgm-R | TTCGCTACCACTGAAATCCAAGAC |
| Psn-F | ATTGCTCCCCGCCATTGCTA |
| Psn-R | CATTGCTCTTACCCTGGTCGCCA |
| hmsH-F | CGTTTCAGTTGCCTGTGTGCTAAC |
| hmsH-R | CATCACTCGGTGTAGACATCGCT |
| YopM-F | CGCATAAAAATTCCCGGCG |
| YopM-R | GCACCTCGAGAATTATGAACGCTCTTGC |
| Oligo 5.1 | GATCCATGCATCATCACCATCACCACTTCATAAATCCAAGAAATGTATCT  AATACTTTTTTGCAAGAACCATTACGTCATTCTTCTAATTTAACTGAGATG |
| Oligo 5.2 | CCGGCATCTCAGTTAAATTAGAAGAATGACGTAATGGTTCTTGCAAAAAA  GTATTAGATACATTTCTTGGATTTATGAAGTGGTGATGGTGATGATGCATG |
