# Supplementary material for: The Yersinia pestis Effector YopM Inhibits Pyrin Inflammasome Activation
Source: PLoS Pathog. 2016 Dec 2;12(12):e1006035. doi: 10.1371/journal.ppat.1006035 (PMC5135138; doi:10.1371/journal.ppat.1006035)
Supplement: S2 Table — (DOCX) [file ppat.1006035.s003.docx]

**Table S2**. Primers used for RT-PCR

| **Primer Name** | **Sequence 5’ to 3’** |
| --- | --- |
| GAPDH F | TGTGTCCGTCGTGGATCTGA |
| GAPDH R | CCTGCTTCACCACCTTCTTGA |
| Pro-IL-1β F | AGGCCACAGGTATTTTGTCG |
| Pro-IL-1β R | GCCCATCCTCTGTGACTCAT |
| Pyrin F | TCATCTGCTAAACACCCTGGA |
| Pyrin R | GGGATCTTAGAGTGGCCCTTC |
