# Supplementary figures and images for: The Yersinia pestis Effector YopM Inhibits Pyrin Inflammasome Activation
Source: PLoS Pathog. 2016 Dec 2;12(12):e1006035. doi: 10.1371/journal.ppat.1006035 (PMC5135138; doi:10.1371/journal.ppat.1006035)

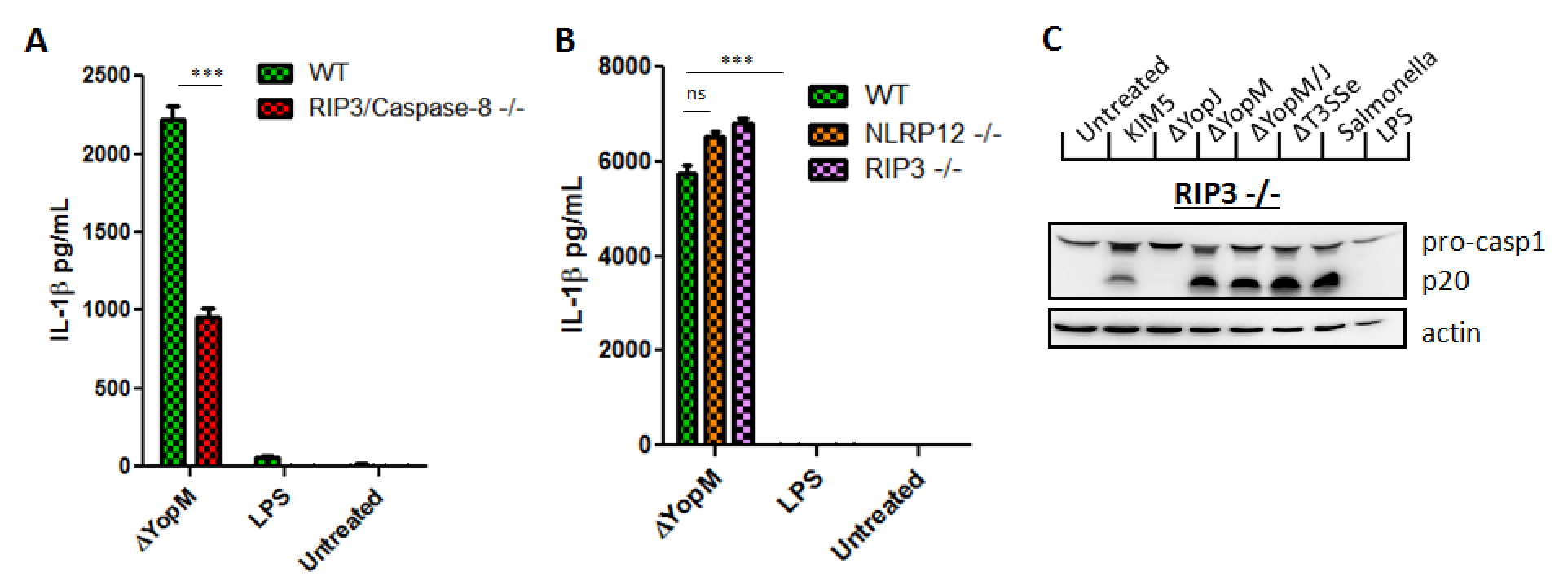

Supplement: S1 Fig — LPS-primed BMDMs were infected with indicated strains of Y. pestis at MOI 10 for 6 hours, and supernatant IL-1β was assayed by ELISA in A) WT, RIP3/Caspase-8 -/- and B) WT, NLRP12 -/-, RIP3 -/-, BMDMs. Decrease of IL-1 release in the absence of caspase-8 cannot be explained by reduced caspase-1 cleavage (Fig 1), but rather reflects reduced transcriptional activity. C) Total protein from LPS-primed RIP3 -/- BMDMs infected with indicated strains (combined cell lysate and supernatant) was separated by SDS-PAGE and analyzed by Western Blot for caspase-1. (TIF) [file ppat.1006035.s004.tif]

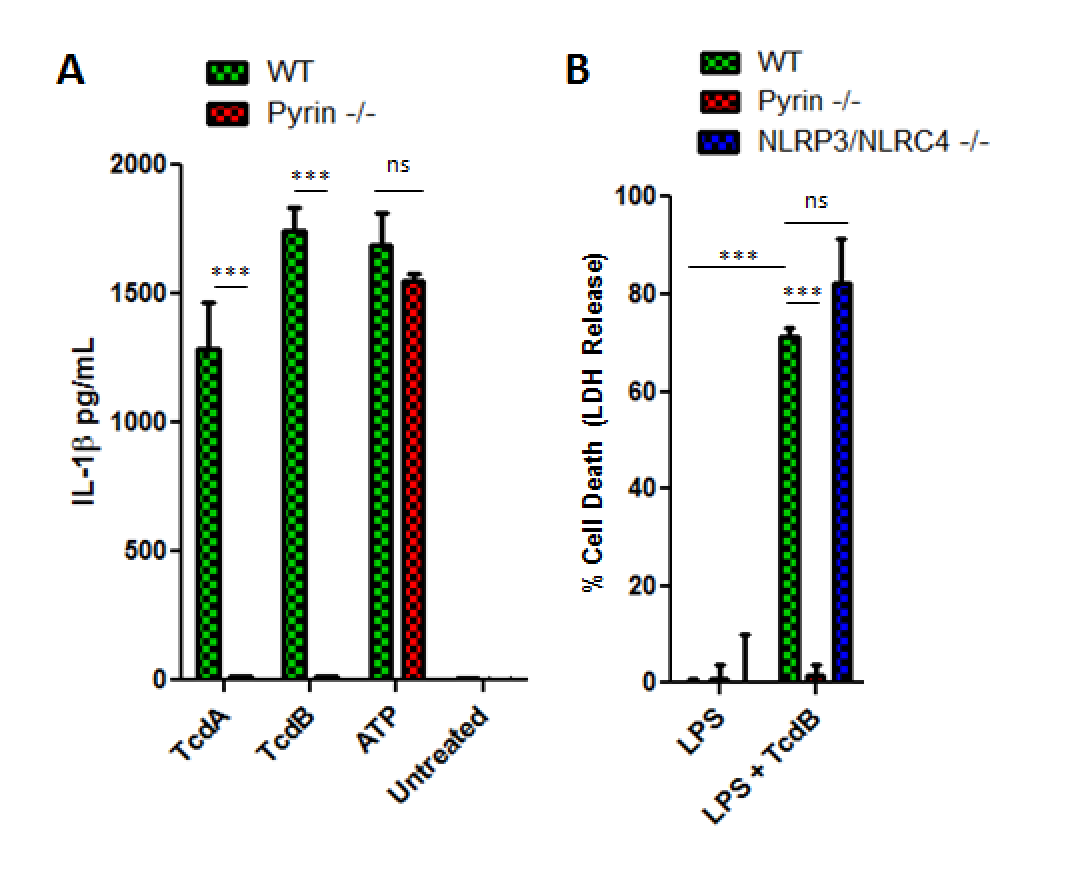

Supplement: S2 Fig — LPS-primed (100 ng/ml) BMDMs of indicated genotypes (WT C57Bl/6 or KO) were treated with 0.2uM TcdA, 0.2uM TcdB, or 5mM ATP. A) supernatant IL-1β was assayed by ELISA and B) cell death was assayed by LDH assay. (TIF) [file ppat.1006035.s005.tif]

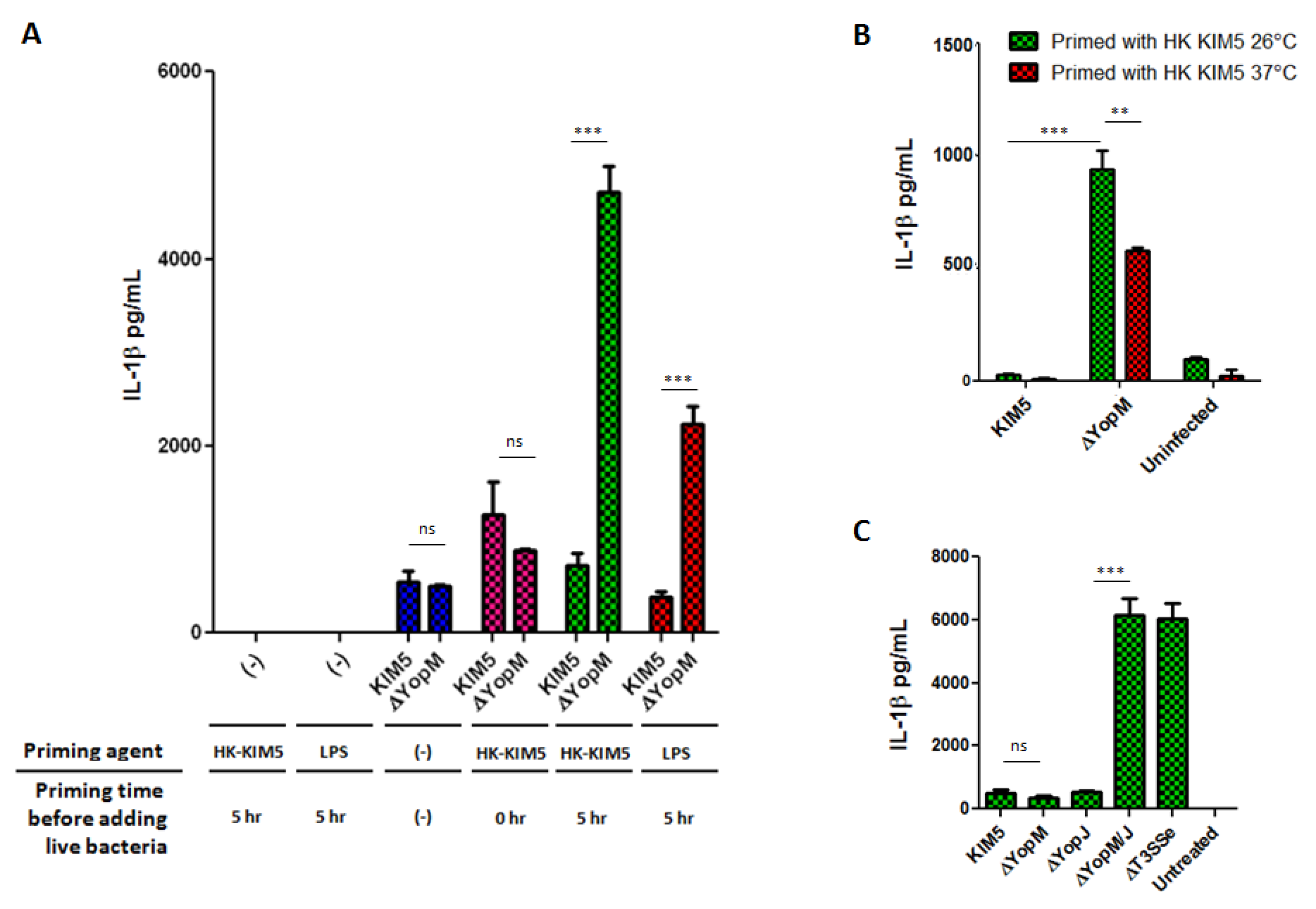

Supplement: S3 Fig — Priming can be achieved with LPS or heat-killed bacteria expressing either hexa- or tetra-acylated LPS. The suppressive action of YopJ appears to contribute to the need for priming. A) 100ng/mL LPS or 1x10^8^ CFU equivalents of heat-killed KIM5 were added to BMDMs either 5 hours before infection, or simultaneously with live KIM5 or ΔYopM at MOI 10. Supernatant from 6 hours p.i. was assayed for IL-1β by ELISA. B) Priming can be achieved with heat-killed Y. pestis regardless of whether it is grown at 26°C or 37°C, despite expression of tetra-acylated LPS with low stimulatory ability. C) Unprimed BMDMs were infected with indicated strains of Y. pestis (temperature-shifted) at MOI 10 for 6 hours, and supernatant IL-1β was assayed by ELISA. It is also worth noting that without priming, KIM5ΔYopM produces IL-1β comparable to parental KIM5, whereas KIM5ΔYopM/J triggers significantly elevated levels of IL-1β (S3 Fig). It is possible that YopJ suppresses priming that occurs during the course of the 6-hour infection, either by inhibiting NF-κB- or MAPK mediated gene expression, or by inducing apoptosis before sufficient priming can occur. This is further suggested by the fact that LPS-priming is not required to elicit a strong IL-1β response with KIM5ΔYopM/J, unlike KIM5ΔYopM where YopJ is present. (TIF) [file ppat.1006035.s006.tif]

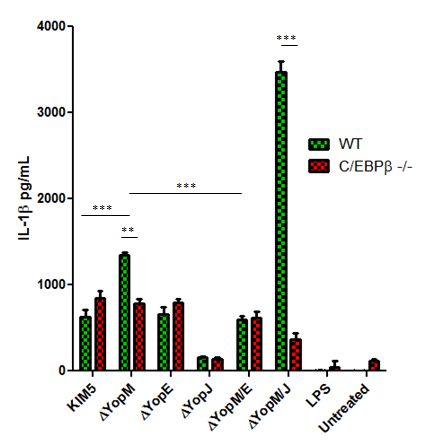

Supplement: S4 Fig — LPS-primed BMDMs were infected with indicated strains of Y. pestis at MOI 10 for 6 hours, and supernatant IL-1β was assayed by ELISA. (TIF) [file ppat.1006035.s007.tif]

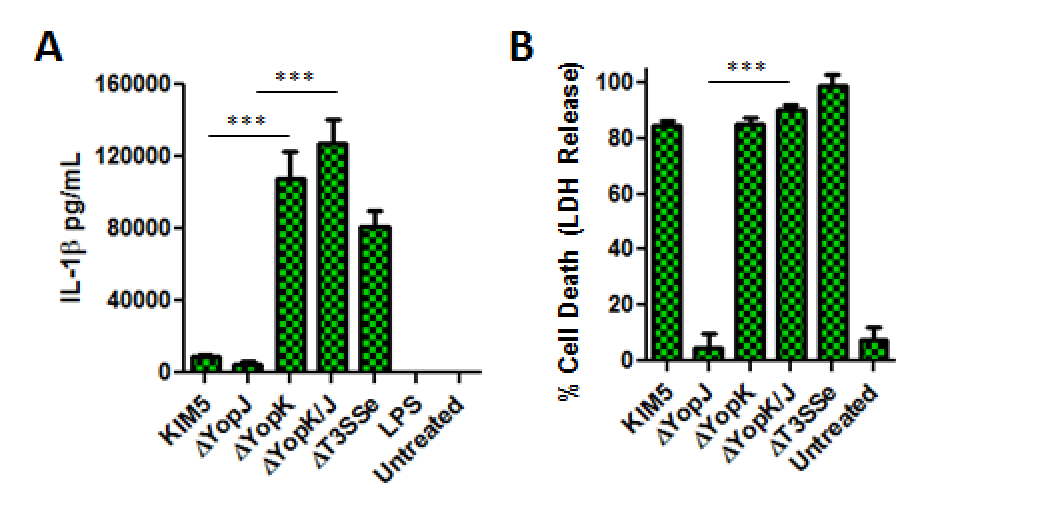

Supplement: S5 Fig — LPS-primed BMDCs were infected with indicated strains of Y. pestis at MOI 10 for 6 hours; A) supernatant IL-1β was assayed by ELISA, and B) cell death was measured by LDH release. (TIF) [file ppat.1006035.s008.tif]
